# A prediction tool for nosocomial multi-drug resistant gram-negative bacilli infections in critically ill patients - prospective observational study

**DOI:** 10.1186/s12879-014-0615-z

**Published:** 2014-11-25

**Authors:** Anupama Vasudevan, Amartya Mukhopadhyay, Jialiang Li, Eugene Goh Yu Yuen, Paul Ananth Tambyah

**Affiliations:** National University Health System, NUHS Tower Block, 1E Kent Ridge Road, Singapore, 119228 Singapore; National University of Singapore, Blk S16, Level 7, 6 Science Drive 2, Singapore, 117546 Singapore

**Keywords:** Nosocomial Infection, Gram-Negative Bacteria, Antibiotic resistance, Intensive care unit, Prediction tool, Bacteremia

## Abstract

**Background:**

The widespread use of empiric broad spectrum antibiotics has contributed to the global increase of Resistant Gram-Negative Bacilli (RGNB) infections in intensive care units (ICU). The aim of this study was to develop a tool to predict nosocomial RGNB infections among ICU patients for targeted therapy.

**Methods:**

We conducted a prospective observational study from August'07 to December'11. All adult patients who were admitted and stayed for more than 24 hours at the medical and surgical ICU's were included. All patients who developed nosocomial RGNB infections 48 hours after ICU admission were identified. A prediction score was formulated by using independent risk factors obtained from logistic regression analysis. This was prospectively validated with a subsequent cohort of patients admitted to the ICUs during the following time period of January-September 2012.

**Results:**

Seventy-six patients with nosocomial RGNB Infection (31bacteremia) were compared with 1398 patients with Systemic Inflammatory Response Syndrome (SIRS) without any gram negative bacterial infection/colonization admitted to the ICUs during the study period. The following independent risk factors were obtained by a multivariable logistic regression analysis - prior isolation of **G**ram negative organism (coeff: 1.1, 95% CI 0.5-1.7); **S**urgery during current admission (coeff: 0.69, 95% CI 0.2-1.2); prior **D**ialysis with end stage renal disease (coeff: 0.7, 95% CI 0.1-1.1); prior use of **C**arbapenems (coeff: 1.3, 95% CI 0.3-2.3) and **S**tay in the ICU for more than 5 days (coeff: 2.4, 95% CI 1.6-3.2). It was validated prospectively in a subsequent cohort (n = 408) and the area-under-the-curve (AUC) of the GSDCS score for predicting nosocomial ICU acquired RGNB infection and bacteremia was 0.77 (95% CI 0.68-0.89 and 0.78 (95% CI 0.69-0.89) respectively. The GSDCS (0-4.3) score clearly differentiated the low (0-1.3), medium (1.4-2.3) and high (2.4-4.3) risk patients, both for RGNB infection (p:0.003) and bacteremia (p:0.009).

**Conclusion:**

GSDCS is a simple bedside clinical score which predicts RGNB infection and bacteremia with high predictive value and differentiates low versus high risk patients. This score will help clinicians to choose appropriate, timely targeted antibiotic therapy and avoid exposure to unnecessary treatment for patients at low risk of nosocomial RGNB infection. This will reduce the selection pressure and help to contain antibiotic resistance in ICUs.

**Electronic supplementary material:**

The online version of this article (doi:10.1186/s12879-014-0615-z) contains supplementary material, which is available to authorized users.

## Background

The increase of drug resistant bacteria worldwide has caused concern amongst healthcare professionals and the wider community [1]. In particular, the increase in resistance among gram negative bacteria over the last decade, has been described as "Bad Bugs, No Drugs" by the Infectious Diseases Society of America [2]. This issue was the key component for the World Health Organization's World Health Day 2011 [3] and major public health action plans have been formulated by international agencies including the United States Centres for Disease Control and Prevention [4] and Public Health England [5].

With their altered bacterial flora, impaired immune response and breached anatomical integrity due to invasive procedures and devices, the intensive care unit (ICU) population is among the highest incidences of nosocomial infections [6],[7]. Many of these nosocomial infections are due to drug resistant bacteria [8] with an increasing predominance of gram negative organisms [9]-[11]. Surveillance studies across the world have demonstrated an increase in resistance among gram negative organisms especially among critically ill patients [12],[13]. In our local setting, third generation cephalosporin-resistant gram negative bacteria (GNB) were found to be the most common organisms among ICU isolates [14].

With few new antibiotics in the pipeline, the emphasis has been on prevention and control of the spread of resistant gram negative bacilli (RGNB) [15]. Effective infection control practices, surveillance measures, antimicrobial stewardship programs [16]-[18] have been implemented to attempt to reduce the occurrence of nosocomial RGNB infections. Widespread inappropriate use of antibiotics in the hospital [19],[20] and ICU [21] is common. This, in addition to the presence of invasive devices, surgical procedures and severe co-morbid conditions are risk factors for RGNB infection and colonization [22]-[25]. Unfortunately, heterogeneity of cohorts, restricted number of risk factors studied and relatively small sample sizes have limited the applicability of many previous studies to direct clinical practice. We believe that it would be useful for clinicians to use these risk factors as an objective bedside tool to start empiric broad-spectrum antibiotic in highly susceptible critically ill patients and avoid antibiotic over-exposure in those at low risk.

In the current study, using clinical, demographic and therapeutic observations, we aim to develop a simple bedside prediction tool for nosocomial RGNB infection in the ICU, in order to help clinicians with selection of empiric antibiotics for patients with SIRS on admission.

## Methods

### Setting

This is a prospective observational cohort study conducted over a period of 4.5 years (August'07- December'11) followed by nine month validation period from January-September'12 at a 1000-bedded tertiary academic medical centre affiliated to the National University of Singapore. All patients aged more than 21 years admitted to the medical and surgical ICUs and stayed for more than 24 hours were included in the study. The Medical ICU (MICU) is a 12 bed unit which admits all patients under the care of internal medicine teams including hematology-oncology but excluding cardiology. The Surgical ICU (SICU) is a 13 bed unit which admits all elective and emergency surgical patients excluding cardiothoracic surgery. Both units are managed by trained intensivists with nursing ratio varying from 1:1 to 1:2 and follow strict infection control practices including WHO recommended hand-hygiene guidelines [26].

### Data collection

A hospital-wide computerized database (Computerized Patient Support System, CPSS, Singapore) [27] that collects the electronic medical records, including discharge summaries and biochemical, hematological, microbiological and radiological investigations, was accessed to record the following data prospectively: demographics, comorbidities, Acute Physiology and Chronic Health Evaluation II (APACHE II) scores on the day of admission to the ICU, surgical interventions, prior hospital admission within one year from the current admission, results of Methicillin-resistant Staphylococcus aureus (MRSA) screening on admission to the ICU, antibiotics usage and, culture and sensitivity of the clinical isolates. For all patients included in the study, detailed antibiotic prescription history was obtained by reviewing their previous electronic records and outpatient medications. The data were collected in a palmtop device using HanDBase4 Database manager (Wellington, FL, USA) and stored in MS Access database for further analysis. All clinical and microbiological details for each patient including the first isolation of GNB from any clinical specimen during the patient's stay in the ICU were recorded. Patients who had a GNB isolated from any of the cultures within 48 hours of admission to the ICU were excluded from the analysis.

### Definitions

#### Resistant gram negative bacilli (RGNB)

*Acinetobacter baumannii, Pseudomonas aeruginosa, Klebsiella pneumoniae, Escherichia coli*: Multi-drug resistance was defined as being non susceptible to > = 1 agent in > = 3 antimicrobial categories. The antimicrobial categories were counted independently for each organism [28].

#### Colonization

Those patients with RGNB/SGNB cultured from any clinical specimen with no clinical signs or symptoms of infection and no treatment initiated or changed by the treating clinician or documented as colonization by the Infectious disease specialist [29].

#### Infection

All patients with RGNB/SGNB cultured from any of the clinical specimens and an infection was documented by the clinicians with initiation or change of treatment for the organism. The criteria for infection are similar to those used by the United States National Health and Safety Network [29]. For those patients with a positive respiratory culture, the quality of the clinical specimen was assessed by calculating the Q score [30] and those positive specimens with unacceptable Q scores were considered to be colonizers. We also calculated the CPIS scores for those patients with acceptable Q scores and only those with a CPIS score of more than 6 were considered to be nosocomial pneumonia [31]. All those patients with confirmed urinary tract infection in our study had more than 10^3^ CFU/ml reported in their culture results.

#### Nosocomial RGNB infection in the ICU

RGNB isolated after 48 hours of admission to the ICU.

#### SIRS

SIRS was confirmed if the patient satisfied 2 or more standard criteria [32].

### Statistical analysis

Analysis was done using STATA 10.1 (STATA Corp, Texas, USA). Patients with nosocomial ICU acquired RGNB infections were compared with SIRS patients who had no GNB isolated during their ICU stay.

*Univariate analysis* was done including all the potential risk factors using Chi-square/Fisher's exact tests for comparing proportions and Student's *t* test/Wilcoxon rank sum tests for continuous variables where applicable.

*Identification of Independent Risk Factors*: Those risk factors with a p ≤ 0.05 in univariate analysis were then included in the forward step wise multivariable logistic regression analysis. The discriminatory power of this derivation model was tested using receiver operating curve (ROC) analysis [33] by assessing the area under the curve (AUC) and the calibration efficiency was tested using Hosmer Lemeshow test [34]. Potential interactions were also checked in the prediction model.

*Score Formulation*: A score was then calculated by assigning points based on the regression coefficients obtained from the logistic regression analysis for the independent risk factors associated with the occurrence of nosocomial RGNB infection in the ICU.

*Prospective validation*: The scores were applied to a separate cohort of patients admitted to the ICUs from January-September 2012. A receiver operating characteristic analysis and area under the curve (AUC) was obtained.

*Assessment of the score in patients with bacteremia*: We tested the score in a subset of RGNB infection with bacteremia using the ROC analysis.

The study was approved by the National Healthcare Group (NHG) Domain Specific Review Board that governs research at our institution (Reference: B/06/140).

## Results

**Figure 1:**
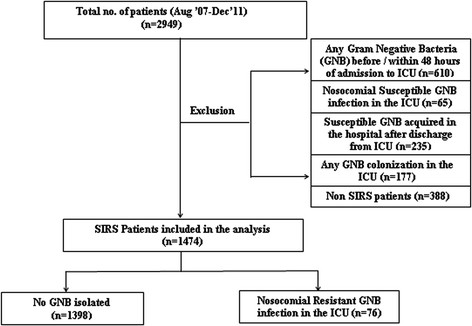
**Patient flowchart.**

Two thousand nine hundred and forty nine patients who stayed for more than 24 hours in the ICU's were included in our study. The details of the patients included for the analysis are shown in Figure 1. Excluding the patients who had a GNB before or within 48 hours of admission to the ICU and those patients with a SGNB during the hospital stay, 1927 patients were included in the derivation cohort. 1474 (76.5%) patients satisfied the criteria for SIRS on admission to the ICU and were included in the risk factor analysis. There were 76 patients with nosocomial RGNB infections in the ICU. The majority of these RGNB infections were bacteremias (40.8%) followed by pneumonia (27.6%) and urinary tract infections (11.8%). Sixteen (22.8%) were polymicrobial RGNB infections and 9 (12%) were polymicrobial bacteremias. The patients with polymicrobial infections were counted as a single infection for the analysis. Table 1 shows the patients characteristics of those with RGNB infection and without GNB infection/colonization.

**Table 1:** **SIRS patients- Nosocomial ICU acquired RGNB Infection and patients with no GNB: Patient characteristics and univariate analysis**

	No GNB* Infection/colonization (n = 1398)	RGNB ^†^Infection (n = 76)	***p*** -value
Gender, n (%)			0.49
*Male*	902 (64.5%)	52 (68.4%)	
*Female*	496 (35.5%)	24 (31.6%)	
Age, years (mean, ±SD)	56.9 (SD17.8)	59.7 (SD17.6)	0.19
APACHEII^‡^ (median, range)	17 (1-55)	19 (2-39)	0.03
Intensive care unit, n (%)			0.02
*Medical*	868 (62.9%)	37 (49.3%)	
*Surgical*	513 (37.1%)	38 (50.7%)	
Comorbidities, n (%)			
*Diabetes Mellitus*	427 (30.5%)	24 (31.6%)	0.85
*Dialysis with end stage renal disease*	130 (9.3%)	14 (18.4%)	0.009
*Peripheral vascular disease*	25 (1.8%)	0 (0%)	0.24
*Cerebrovascular accident*	207 (14.8%)	8 (10.5%)	0.3
*Peptic ulcer disease*	67 (4.8%)	4 (5.3%)	0.85
*Myocardial Infarction*	178 (12.7%)	10 (13.2%)	0.91
*Congestive cardiac failure*	48 (3.4%)	4 (5.3%)	0.4
*Liver disease*	52 (3.7%)	3 (3.9%)	0.92
*Leukemia*	34 (2.4%)	4 (5.3%)	0.13
*Malignancy*	163 (11.7%)	9 (11.8%)	0.96
Procedure/treatment, n (%)			
*Mechanical ventilation*	1310 (93.7%)	75 (98.7%)	0.08
*Duration of mechanical ventilation*	4.5 (SD 4.02)	7.5 (SD 4.9)	0.01
*Central venous catheter*	920 (65.8%)	58 (76.3%)	0.05
*Intra-arterial line*	1113 (79.6%)	65 (89.5%)	0.21
*Urinary catheter*	1156 (82.7%)	70 (92.1%	0.04
*Surgery this admission before RGNB*	524 (37.5%)	42 (55.3%)	0.002
*Transfusion*	411 (29.4%)	35 (46.1%)	0.02
*Sedation*	982 (81%)	67 (91.8%)	0.02
*Carbapenems within 6 months*	409 (29.3%)	39 (51.3%)	<0.001
*3rdGeneration Cephalosporins within 6 months*	721 (51.6%)	34 (44.7%)	0.25
*Other Cephalosporins within 6 months*	200 (14.3%)	17 (22.7%)	0.05
*Quinolones within 6 months*	248 (17.7%)	17 (22.7%)	0.28
*Penicillins within 6 months*	385 (27.5%)	29 (38.7%)	0.04
*Aminoglycosides within 6 months*	92 (6.6%)	11 (14.7%)	0.007
*Augmentin/Unasyn within 6 months*	615 (43.9%)	24 (32%)	0.04
*Other antibiotics within 6 months*	790 (56.5%)	52 (69.3%)	0.03
*Hospitalization in the past one year*	246 (17.6%)	19 (25%)	0.1
*Median days of pre-ICU stay in the hospital (range)*	0 (0-40)	0 (0-29)	0.05
*Days of stay in ICU >5*	444 (31.8%)	59 (77.6%)	<0.001
Cultures, n (%)			
*Positive MRSA* ^ll^ *screening on admission to ICU*	69 (5.4%)	5 (7.1%)	0.53
*Presence of any GNB within 6 months*	102 (7.3%)	23 (30.3%)	<0.001

*Gram negative bacteria, ^†^Resistant gram negative bacteria, ^‡^Acute Physiology and Chronic Health Evaluation, ^ll^Methicillin-resistant Staphylococcus aureus.

### Identification of independent risk factors

By including the risk factors with a *p* ≤0.05 from the univariate analysis (Table 1) in a forward logistic regression analysis, we obtained the independent risk factors for nosocomial RGNB infection (Table 2). This prediction model had a hosmer-lemeshow fit of 0.63 and an area under the curve of 0.80 (95% CI: 0.75-0.85).

**Table 2:** Nosocomial ICU acquired RGNB* Infection: Independent risk factors- logistic regression (Comparison with SIRS patients with no GNB ^**†**^ **Infection/Colonization)**

Nosocomial ICU acquired RGNB infection	Coef.	P > z	95% Conf. interval	
*Days of stay in ICU >5*	2.37	<0.001	1.56	3.18
*Carbapenems within 6 months*	1.32	0.008	0.34	2.30
*Presence of any GNB within 6 months*	1.14	<0.001	0.58	1.70
*Dialysis with end stage renal disease*	0.79	0.017	0.14	1.45
*Surgery this admission before RGNB*	0.69	0.005	0.21	1.19
Interaction: Carbapenems *days in ICU > 5	-1.42	0.013	-2.55	-0.30
_cons	-5.02	<0.001	-5.78	-4.26

*Resistant gram negative bacteria, ^†^Gram negative bacteria.

### Score formulation

Based on the regression coefficients from the logistic regression (Table 2), we formulated the GSDCS (**G**ram Negative bacteria in last 6 months, **S**urgery during current admission before RGNB, prior **D**ialysis with end stage renal disease, prior use of **C**arbapenem within last 6 months, **S**tay in the ICU for more than 5 days) score by allotting the points as follows: 1 point each for presence of prior GNB and prior administration of carbapenems within 6 months, 0.6 points for surgery before RGNB, 0.7 points for dialysis with end stage renal disease and 2 points for a stay of more than 5 days in the ICU. All these individual points were added up to achieve the score. In order to factor in the interaction, a score of -1 was added for all those patients who had stayed for more than 5 days in the ICU with prior exposure to Carbapenem to obtain the final score for prediction of nosocomial RGNB infection in the ICU. The sensitivity and specificity values at the different cut-off points are shown in Table 3. The patients were then segregated into low (0-1.3 points), medium (1.4-2.3 points) and high risk (2.4-4.3 points) categories based on their scores. The prevalence of RGNB infection among the three groups in the increasing order were 1.2%, 6.3% and 19.8% respectively (p < 0.001).

**Table 3:** **Sensitivity and Specificity values of the scores**

Cutpoint	Sensitivity	Specificity	Correctly classified	LR+	LR-
(> = 0)	100.00%	0.00%	5.16%	1	
(> = .6)	94.74%	28.97%	32.36%	1.3338	0.1817
(> = .7)	92.11%	48.64%	50.88%	1.7934	0.1623
(> = 1)	92.11%	51.79%	53.87%	1.9104	0.1524
(> = 1.3)	86.84%	59.87%	61.26%	2.1641	0.2198
(> = 1.6)	85.53%	61.02%	62.28%	2.1939	0.2372
(> = 1.7)	69.74%	72.39%	72.25%	2.5257	0.4181
(> = 2)	68.42%	73.89%	73.61%	2.6206	0.4274
(> = 2.2)	60.53%	82.62%	81.48%	3.4821	0.4778
(> = 2.3)	52.63%	87.41%	85.62%	4.1806	0.5419
(> = 2.6)	51.32%	88.70%	86.77%	4.5405	0.5489
(> = 2.7)	32.89%	95.35%	92.13%	7.0749	0.7038
(> = 2.9)	28.95%	96.35%	92.88%	7.935	0.7374
(> = 3)	25.00%	96.92%	93.22%	8.1279	0.7738
(> = 3.2)	22.37%	97.78%	93.89%	10.0874	0.7939
(> = 3.3)	13.16%	98.78%	94.37%	10.8204	0.8791
(> = 3.6)	9.21%	99.21%	94.57%	11.7057	0.9151
(> = 3.9)	2.63%	99.79%	94.78%	12.2632	0.9758
(> = 4.3)	0.00%	99.93%	94.78%	0	1.0007
(>4.3)	0.00%	100.00%	94.84%		1

There were 31 (40.8%) patients with bacteremia among the patients with RGNB infections. The GSDCS score yielded an AUC of 0.83 (95% CI 0.76-0.89) when applied to bacteremic patients. The prevalence of RGNB bacteremia in the low, medium and high risk categories was 0.2%, 3% and 9.7% respectively (p < 0.001).

### Prospective validation

**Figure 2:**
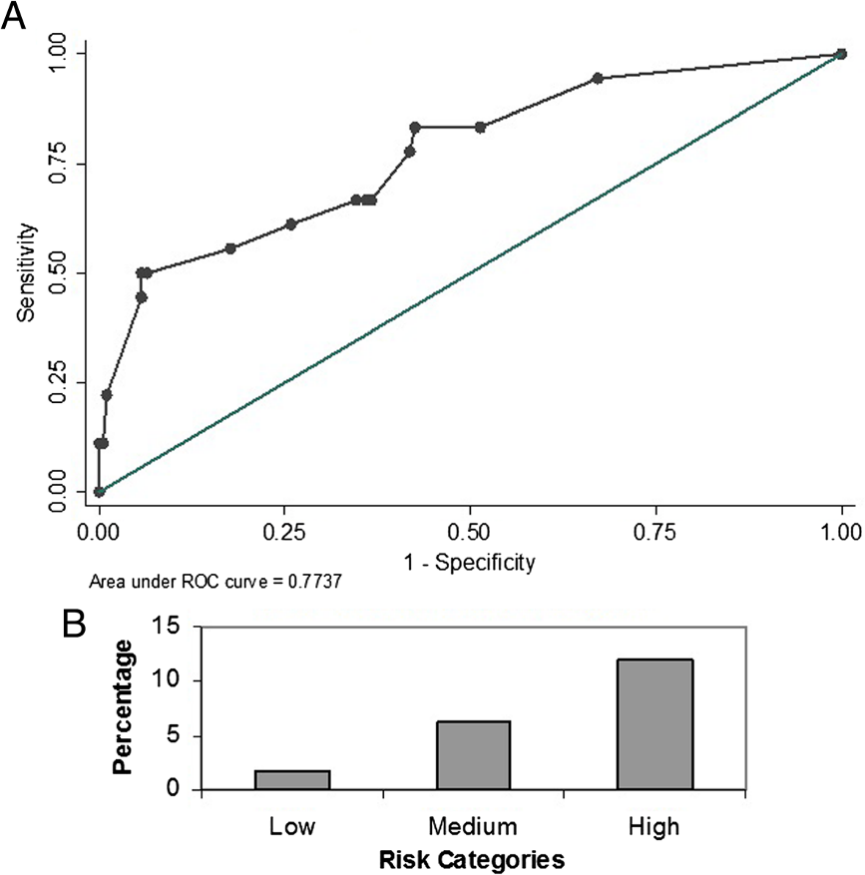
**Performance of GSDCS score in predicting nosocomial RGNB infection. A**: Receiver Operating Characteristics. **B**: Prevalence by risk categories.

**Figure 3:**
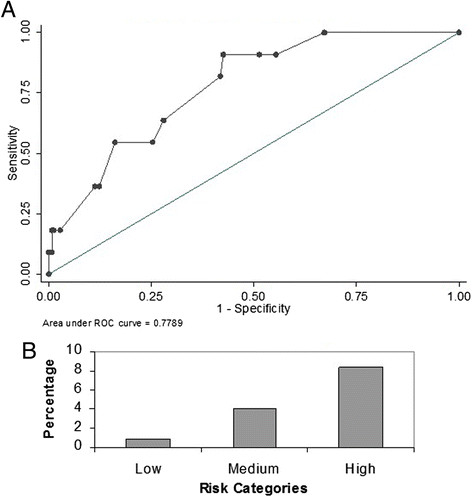
**Performance of GSDCS score in predicting nosocomial RGNB bacteremia: A: Receiver operating characteristics.** **B**: prevalence by risk categories.

The score was then applied to a new cohort of patients admitted to both the ICUs from January - September 2012. Similar to the derivation cohort, we excluded all those patients with a positive GNB culture before or within 2 days of ICU admission. There were 483 patients who were admitted during the validation period and 64 of them had a GNB isolated before or within 48 hours of admission to the ICU and were excluded from the analysis. Of the remaining 419 patients, 408 satisfied the criteria for SIRS and were included in the validation cohort. 18 of these 408 patients had nosocomial ICU acquired RGNB infection. The GSDCS score yielded an AUC of 0.77 (95% CI 0.68-0.89) for prediction of nosocomial RGNB infection in the ICU (Figure 2A). The prevalence of RGNB infections among patients with low, medium and high risk categories were 1.7%, 6.3% and 12% respectively (p0.003) (Figure 2B). Eleven (61.1%) of the validation cohort with RGNB infections had bacteremia. The GSDCS score yielded an AUC of 0.78 (95% CI 0.69-0.89) when applied to predict RGNB bacteremia among this cohort (Figure 3A). The prevalence of RGNB bacteremia was 0.9%, 4% and 8% among the low, medium and high risk categories (p 0.009) (Figure 3B).

## Discussion

In our setting, prior isolation of any GNB, receipt of carbapenems in the previous 6 months, surgery, patients with end stage renal disease undergoing dialysis and those with an ICU stay of more than 5 days were associated with a higher risk of contracting an RGNB infection in the ICU. We found that a negative interaction existed between carbapenem administration and a stay of more than 5 days in the ICU. In our cohort, we found that end stage renal failure on dialysis was more important than the overall APACHEII score in the multivariable analysis. This is similar to others who have found that APACHE II score does not necessarily predict the risk of infection with multi-drug resistant organisms in the ICU [23],[35] Based on the risk factors identified, we formulated the bedside GSDCS score (0-4.3) in line with Wasson et al. [36].

When applied to the validation cohort of patients from same ICUs, the score yielded AUC's of 0.77 and 0.78 for RGNB infection and bacteremia respectively. The score was able to clearly segregate the low from high risk patients, for both RGNB infection and bacteremia.

With their reduced immune status and increased antibiotic usage in ICUs, critically ill patients are vulnerable to nosocomial infections [37]. The American Thoracic Society guideline [38] recommends early and appropriate use of antibiotics to reduce morality in hospital acquired infections. Delayed initiation of appropriate antibiotics [39] or changing the antibiotics based on the susceptibility results available later in the course of treatment [40],[41] may be associated with increased mortality among patients with hospital acquired pneumonia. Given the pressing need to get the antibiotic treatment "right the first time, every time and without delay", the broadest spectrum antibiotics have become the first line of therapy for nosocomial infections in many modern ICUs. A study involving 43 Italian ICUs showed that 75% of ICU patients *without* sepsis received antibiotics with no reason identified for 20% of them and "prophylaxis" as the reason in the majority [42]. This leads to widespread, over-usage of antibiotics with concomitant "collateral damage" in terms of selection of resistant organisms. In this context, we have shown before that even a short duration of carbapenem use in critically ill patients increases the risk of infection or colonization with multidrug resistant bacteria [43]. At the same time, inadequate definitive antimicrobial therapy is consistently associated with increased mortality in critically ill patients mainly due to the presence of resistant organisms [44]. There is therefore a critical need to identify, at the bedside, which patients are at highest risk for nosocomial infection with multi-drug resistant pathogens so that initial empiric therapy can be targeted at these patients without adversely affecting the rest of the patients in the ICU.

Unfortunately, many previous studies which identified risk factors for RGNB in ICU [22],[23],[45]-[47] were either limited by their retrospective study design or by focusing on specific bacteria, drugs or groups of patients. Further, without aggregating these risk factors, the utility of this information at the bedside is limited. Comprehensive decision analysis tools and scoring systems have been employed to overcome some of these issues. One such widely used tool is the clinical pulmonary infection score (CPIS) proposed by Pugin et al. for Ventilator Associated Pneumonia although not specifically targeting antibiotic resistant pathogens [48]. With the limitations of any decision analysis tool, CPIS has been used for prognostication [49] and most importantly, to reduce indiscriminate antibiotic usage [50]. Among patients with health-care associated pneumonia, Shorr AF et al. developed a prediction tool using 4 criteria to identify patients with higher risk of acquiring antibiotic resistant bacteria - these include recent hospitalization, nursing home residence, hemodialysis and ICU admission [51]. Similarly, a prediction tool was developed for predicting multidrug resistance in P.*aerugionsa* [52] among patients with respiratory tract infections using a case control methodology. Although informative, neither was able to generate a comprehensive validated scoring system for RGNB infection/bacteremia which could be applied at the bedside in the ICUs. Since application of clinical evidence varies tremendously amongst clinicians [53], use of a simple scoring system would help narrow the variability between clinicians in decision making especially in the complex ICU setting.

Our GSDCS score is easy to apply and may help clinicians in ICU to identify the patients at risk of RGNB infection and bacteremia who need early broad-spectrum appropriate antibiotics while reducing the risk of unnecessary antibiotic exposures. Striking this balance will be crucial to reduce the emergence of antimicrobial resistance while averting mortality from nosocomial infections.

Strengths of this current study include the use of readily available bedside information to formulate the GSDCS score. The prospective validation of the score using a separate cohort indicated that score was indeed robust in predicting a nosocomial drug resistant gram negative infection. This study is a prospective observational cohort study and all the patients were followed up diligently until their discharge from the hospital. No changes were instituted in the treatment plan as this study was conducted by a research assistant independent of the treating team. However, our study also had some limitations. This was a single centre study and further validation needs to be done in other settings. We also excluded patients who had RGNB cultured before or with 48 hours of ICU admission to capture our primary outcome of interest - patients who developed ICU acquired nosocomial infections. Because of this, our numerator was relatively small in spite of screening a very large number of ICU admissions. There was no routine screening done for RGNB during the study period as unlike MRSA, there are no universally accepted screening methods for RGNB. We were also unable to obtain the comprehensive past antibiotic history of those patients with previous admission to different hospital systems if any.

## Conclusion

This simple prospectively validated risk stratification score for prediction of nosocomial RGNB infection and bacteremia will help the clinicians to identify critically ill patients who are at risk of antibiotic resistant gram-negative infections. This should logically lead to targeted antibiotic treatment while avoiding antibiotic overuse which worsens the vicious cycle of resistance in the ICU.

## Authors' contributions

Conception and design: AM, GEY,PAT. Data acquisition: AV, GEY. Analysis & interpretation: AV, AM, JLL, PAT. Drafting the manuscript for important intellectual content: AV, AM, JLL, PAT. Final approval of the submitted version: AV, AM, JLL, PAT. Agreement to be accountable for all aspects of work: AV, AM, JLL, PAT. Unfortunately Dr. Eugene passed away during the drafting and final stages of work. All authors read and approved the final manuscript.

## Authors' information

AV: BDS, MPH. Doctorate student at National University of Singapore.

AM: FRCP. Senior Consultant, Respiratory and Critical Care Medicine, National University Health System.

JLL: PhD. Associate Professor, Statistics. National University of Singapore.

PAT: MD. Senior Consultant & Professor, National University Health System.

## Authors’ original submitted files for images

Below are the links to the authors’ original submitted files for images. Authors’ original file for figure 1 Authors’ original file for figure 2 Authors’ original file for figure 3 Authors’ original file for figure 4 Authors’ original file for figure 5

## Abbreviations

ICU: Intensive Care Unit
GNB: Gram Negative Bacteria
RGNB: Resistant Gram Negative Bacteria
SGNB: Susceptible Gram Negative Bacteria
MICU: Medical Intensive Care Unit
SICU: Surgical Intensive Care Unit
APACHE: Acute Physiology and Chronic Health Evaluation
MRSA: Methicillin-resistant Staphylococcus aureus
SIRS: Systemic Inflammatory Response Syndrome
ROC: Receiver operating curve
AUC: Area Under the Curve
OR: Odds ratios
CPIS: Clinical pulmonary infection score
HL: Hosmer Lemeshow
CI: Confidence Interval

## References

[1] Wise R, Hart T, Cars O, Streulens M, Helmuth R, Huovinen P, Sprenger M (1998). Antimicrobial resistance. Is a major threat to public health. BMJ.

[2] Boucher HW, Talbot GH, Bradley JS, Edwards JE, Gilbert D, Rice LB, Scheld M, Spellberg B, Bartlett J (2009). Bad bugs, no drugs: no ESKAPE! An update from the Infectious Diseases Society of America. Clin Infect Dis.

[3] World Health Day: Media Fact Sheet, Antimicrobial resistance: no action today, no cure tomorrow [], [http://www.cdc.gov/media/releases/2011/f0407_antimicrobialresistance.pdf]

[4] Integrated Task Force on Antimicrobial Resistance: A public health action plan to combat antimicrobial resistance [], [http://www.cdc.gov/drugresistance/pdf/public-health-action-plan-combat-antimicrobial-resistance.pdf]

[5] European Centre for Disease Prevention and Control: *ECDC Strategic Multi-Annual Programme 2007-2013: Public Health Activities, Disease-Specific Programmes and MultilateralPartnerships.* ECDC; 2008:48 [], [http://www.ecdc.europa.eu/en/aboutus/Key%20Documents/07-13_KD_Strategic_multiannual_programme.pdf]

[6] Donowitz LG, Wenzel RP, Hoyt JW (1982). High risk of hospital-acquired infection in the ICU patient. Crit Care Med.

[7] Vincent JL, Bihari DJ, Suter PM, Bruining HA, White J, Nicolas-Chanoin MH, Wolff M, Spencer RC, Hemmer M (1995). The prevalence of nosocomial infection in intensive care units in Europe. Results of the European Prevalence of Infection in Intensive Care (EPIC) Study. EPIC International Advisory Committee. JAMA.

[8] Flaherty JP, Weinstein RA (1996). Nosocomial infection caused by antibiotic-resistant organisms in the intensive-care unit. Infect Control Hosp Epidemiol.

[9] Van Duijn PJ, Dautzenberg MJD, Oostdijk EAN (2011). Recent trends in antibiotic resistance in European ICUs. Curr Opin Crit Care.

[10] European Centre for Disease Prevention and Control: Antimicrobial resistance surveillance in Europe 2009. Annual Report of the European Antimicrobial Resistance Surveillance Network (EARS-Net). 2010, [], [http://www.ecdc.europa.eu/en/publications/Publications/1011_SUR_annual_EARS_Net_2009.pdf]

[11] Rosenthal VD, Maki DG, Mehta A, Alvarez-Moreno C, Leblebicioglu H, Higuera F, Cuellar LE, Madani N, Mitrev Z, Dueñas L, Navoa-Ng JA, Garcell HG, Raka L, Hidalgo RF, Medeiros EA, Kanj SS, Abubakar S, Nercelles P, Pratesi RD (2008). International Nosocomial Infection Control Consortium report, data summary for 2002-2007, issued January 2008. Am J Infect Control.

[12] National Nosocomial Infections Surveillance (NNIS) System Report, data summary from January 1992 through June 2004, issued October 2004. Am J Infect Control. 2004, 32: 470-485. 10.1016/j.ajic.2004.10.001. 10.1016/S0196655304005425 15573054

[13] Chung DR, Song J-H, Kim SH, Thamlikitkul V, Huang S-G, Wang H, So TM-K, Yasin RMD, Hsueh P-R, Carlos CC, Hsu LY, Buntaran L, Lalitha MK, Kim MJ, Choi JY, Kim SI, Ko KS, Kang C-I, Peck KR (2011). High prevalence of multidrug-resistant nonfermenters in hospital-acquired pneumonia in Asia. Am J Respir Crit Care Med.

[14] Hsu L-Y, Tan T-Y, Jureen R, Koh T-H, Krishnan P, Tzer-Pin Lin R, Wen-Sin Tee N, Tambyah PA (2007). Antimicrobial drug resistance in Singapore hospitals. Emerg Infect Dis.

[15] Carlet J, Jarlier V, Harbarth S, Voss A, Goossens H, Pittet D: Ready for a world without antibiotics? The Pensières antibiotic resistance call to action. *Antimicrob Resist Infect Control* 2012, 1:11., 10.1186/2047-2994-1-11 PMC3436635 22958833

[16] Kollef MH, Golan Y, Micek ST, Shorr AF, Restrepo MI (2011). Appraising contemporary strategies to combat multidrug resistant gram-negative bacterial infections--proceedings and data from the Gram-Negative Resistance Summit. Clin Infect Dis.

[17] McGowan JE (2006). Resistance in nonfermenting gram-negative bacteria: multidrug resistance to the maximum. Am J Med.

[18] Dortch MJ, Fleming SB, Kauffmann RM, Dossett LA, Talbot TR, May AK (2011). Infection reduction strategies including antibiotic stewardship protocols in surgical and trauma intensive care units are associated with reduced resistant gram-negative healthcare-associated infections. Surg Infect (Larchmt).

[19] Lautenbach E, Larosa LA, Kasbekar N, Peng HP, Maniglia RJ, Fishman NO (2003). Fluoroquinolone utilization in the emergency departments of academic medical centers: prevalence of, and risk factors for, inappropriate use. Arch Intern Med.

[20] Hecker MT, Aron DC, Patel NP, Lehmann MK, Donskey CJ (2003). Unnecessary use of antimicrobials in hospitalized patients: current patterns of misuse with an emphasis on the antianaerobic spectrum of activity. Arch Intern Med.

[21] Erbay A, Bodur H, Akinci E, Colpan A (2005). Evaluation of antibiotic use in intensive care units of a tertiary care hospital in Turkey. J Hosp Infect.

[22] Alexiou VG, Michalopoulos A, Makris GC, Peppas G, Samonis G, Falagas ME (2012). Multi-drug-resistant gram-negative bacterial infection in surgical patients hospitalized in the ICU: a cohort study. Eur J Clin Microbiol Infect Dis.

[23] Kim SY, Jung JY, Kang YA, Lim JE, Kim EY, Lee SK, Park SC, Chung KS, Park BH, Kim YS, Kim SK, Chang J, Park MS (2012). Risk factors for occurrence and 30-day mortality for carbapenem-resistant Acinetobacter baumannii bacteremia in an intensive care unit. J Korean Med Sci.

[24] Tumbarello M, Repetto E, Trecarichi EM, Bernardini C, De Pascale G, Parisini A, Rossi M, Molinari MP, Spanu T, Viscoli C, Cauda R, Bassetti M (2011). Multidrug-resistant Pseudomonas aeruginosa bloodstream infections: risk factors and mortality. Epidemiol Infect.

[25] Varley AJ, Williams H, Fletcher S (2009). Antibiotic resistance in the intensive care unit. Contin Educ Anaesthesia, Crit Care Pain.

[26] WHO Guidelines on Hand Hygiene in Health Care: First Global Patient Safety Challenge Clean Care Is Safer Care. 2009 PubMed-NCBI [], [http://www.ncbi.nlm.nih.gov/pubmed/23805438] 23805438

[27] Ong BKC (2002). Leveraging on information technology to enhance patient care: a doctor's perspective of implementation in a Singapore academic hospital. Ann Acad Med Singapore.

[28] Magiorakos A-P, Srinivasan A, Carey RB, Carmeli Y, Falagas ME, Giske CG, Harbarth S, Hindler JF, Kahlmeter G, Olsson-Liljequist B, Paterson DL, Rice LB, Stelling J, Struelens MJ, Vatopoulos A, Weber JT, Monnet DL (2012). Multidrug-resistant, extensively drug-resistant and pandrug-resistant bacteria: an international expert proposal for interim standard definitions for acquired resistance. Clin Microbiol Infect.

[29] Garner JS, Jarvis WR, Emori TG, Horan TC, Hughes JM (1988). CDC definitions for nosocomial infections, 1988. Am J Infect Control.

[30] Vasudevan A, Chuang L, Jialiang L, Mukhopadhyay A, Goh EY-Y, Tambyah PA (2013). Inappropriate empirical antimicrobial therapy for multidrug-resistant organisms in critically ill patients with pneumonia is not an independent risk factor for mortality: results of a prospective observational study of 758 patients. J Glob Antimicrob Resist.

[31] Rotstein C, Evans G, Born A, Grossman R, Light RB, Magder S, McTaggart B, Weiss K, Zhanel GG (2008). Clinical practice guidelines for hospital-acquired pneumonia and ventilator-associated pneumonia in adults. Can J Infect Dis Med Microbiol.

[32] Bone RC: Definitions for sepsis and organ failure and guidelines for the use of innovative therapies in sepsis. The ACCP/SCCM Consensus Conference Committee. American College of Chest Physicians/Society of Critical Care Medicine. *Chest J* 1992, 101:1644., 10.1378/chest.101.6.1644 1303622

[33] Lasko TA, Bhagwat JG, Zou KH, Ohno-Machado L (2005). The use of receiver operating characteristic curves in biomedical informatics. J Biomed Inform.

[34] Hosmer DW, Lemenshow S (2000). Applied logistic regression. 2nd.

[35] Akinci E, Colpan A, Bodur H, Balaban N, Erbay A (2005). Risk factors for ICU-acquired imipenem-resistant Gram-negative bacterial infections. J Hosp Infect.

[36] Wasson JH, Sox HC, Neff RKGL (1985). Clinical prediction rules. Applications and methodological standards. N Engl J Med.

[37] Brusselaers N, Vogelaers D, Blot S: The rising problem of antimicrobial resistance in the intensive care unit. *Ann Intensive Care* 2011, 1:47., 10.1186/2110-5820-1-47 PMC3231873 22112929

[38] Guidelines for the management of adults with hospital-acquired, ventilator-associated, and healthcare-associated pneumonia. Am J Respir Crit Care Med. 2005, 171: 388-416. 10.1164/rccm.200405-644ST. 10.1164/rccm.200405-644ST 15699079

[39] Iregui M, Ward S, Sherman G, Fraser VJ, Kollef MH (2002). Clinical importance of delays in the initiation of appropriate antibiotic treatment for ventilator-associated pneumonia. Chest.

[40] Kollef MH, Ward S (1998). The influence of mini-BAL cultures on patient outcomes: implications for the antibiotic management of ventilator-associated pneumonia. Chest.

[41] Álvarez-Lerma F, Grau S (2012). Management of antimicrobial use in the intensive care unit. Drugs.

[42] Malacarne P, Rossi C, Bertolini G (2004). Antibiotic usage in intensive care units: a pharmaco-epidemiological multicentre study. J Antimicrob Chemother.

[43] Donaldson AD, Razak L, Liang LJ, Fisher DA, Tambyah PA (2009). Carbapenems and subsequent multiresistant bloodstream infection: does treatment duration matter?. Int J Antimicrob Agents.

[44] Ibrahim EH, Sherman G, Ward S, Fraser VJ, Kollef MH (2000). The influence of inadequate antimicrobial treatment of bloodstream infections on patient outcomes in the ICU setting. Chest.

[45] Playford EG, Craig JC, Iredell JR (2007). Carbapenem-resistant Acinetobacter baumannii in intensive care unit patients: risk factors for acquisition, infection and their consequences. J Hosp Infect.

[46] Pop-Vicas A, Strom J, Stanley K, D'Agata EMC (2008). Multidrug-resistant gram-negative bacteria among patients who require chronic hemodialysis. Clin J Am Soc Nephrol.

[47] Routsi C, Pratikaki M, Platsouka E, Sotiropoulou C, Papas V, Pitsiolis T, Tsakris A, Nanas S, Roussos C (2013). Risk factors for carbapenem-resistant Gram-negative bacteremia in intensive care unit patients. Intensive Care Med.

[48] Pugin J, Auckenthaler R, Mili N, Janssens JP, Lew PD, Suter PM (1991). Diagnosis of ventilator-associated pneumonia by bacteriologic analysis of bronchoscopic and nonbronchoscopic "blind" bronchoalveolar lavage fluid. Am Rev Respir Dis.

[49] Luna CM, Blanzaco D, Niederman MS, Matarucco W, Baredes NC, Desmery P, Palizas F, Menga G, Rios F, Apezteguia C (2003). Resolution of ventilator-associated pneumonia: prospective evaluation of the clinical pulmonary infection score as an early clinical predictor of outcome. Crit Care Med.

[50] Singh N, Rogers P, Atwood CW, Wagener MM, Yu VL (2000). Short-course empiric antibiotic therapy for patients with pulmonary infiltrates in the intensive care unit. A proposed solution for indiscriminate antibiotic prescription. Am J Respir Crit Care Med.

[51] Shorr AF, Zilberberg MD, Micek ST, Kollef MH (2008). Prediction of infection due to antibiotic-resistant bacteria by select risk factors for health care-associated pneumonia. Arch Intern Med.

[52] Lodise TP, Miller CD, Graves J, Furuno JP, McGregor JC, Lomaestro B, Graffunder E, McNutt L-A (2007). Clinical prediction tool to identify patients with Pseudomonas aeruginosa respiratory tract infections at greatest risk for multidrug resistance. Antimicrob Agents Chemother.

[53] McGinn T, Jervis R, Wisnivesky J, Keitz S, Wyer PC (2008). Tips for teachers of evidence-based medicine: clinical prediction rules (CPRs) and estimating pretest probability. J Gen Intern Med.

