# Supplementary figures and images for: A prediction tool for nosocomial multi-drug resistant gram-negative bacilli infections in critically ill patients - prospective observational study
Source: BMC Infect Dis. 2014 Nov 25;14:615. doi: 10.1186/s12879-014-0615-z (PMC4252002; doi:10.1186/s12879-014-0615-z)

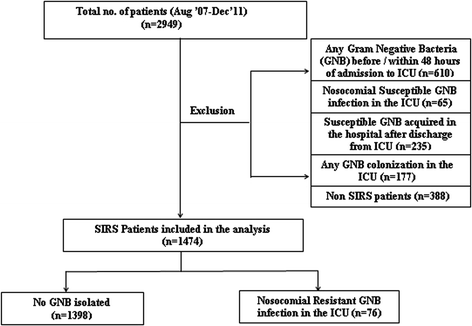

Supplement: Supplementary file 1 — Authors’ original file for figure 1 [file 12879_2014_615_MOESM1_ESM.gif]

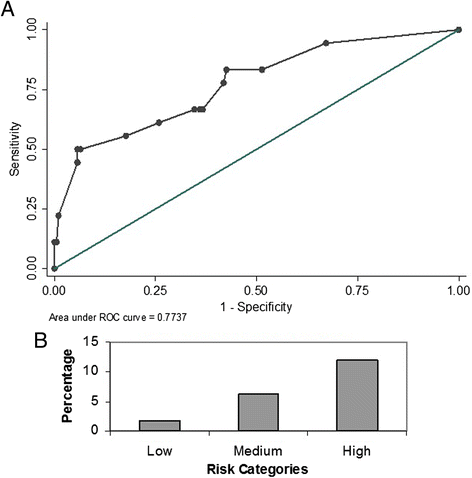

Supplement: Supplementary file 2 — Authors’ original file for figure 2 [file 12879_2014_615_MOESM2_ESM.gif]

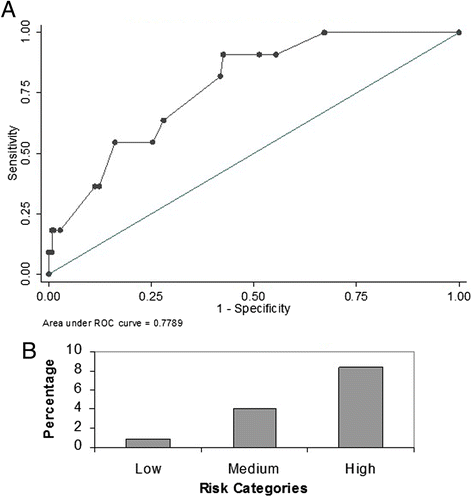

Supplement: Supplementary file 3 — Authors’ original file for figure 3 [file 12879_2014_615_MOESM3_ESM.gif]

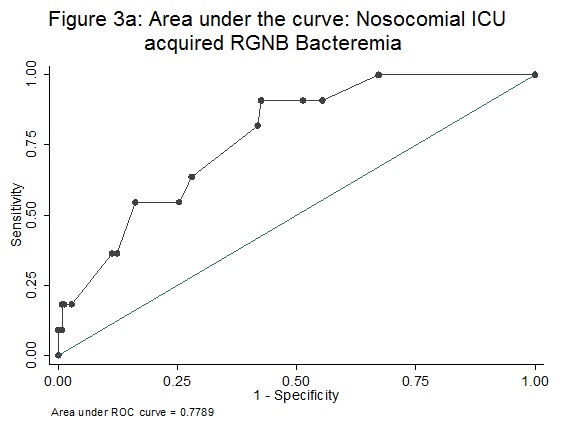

Supplement: Supplementary file 4 — Authors’ original file for figure 4 [file 12879_2014_615_MOESM4_ESM.jpeg]

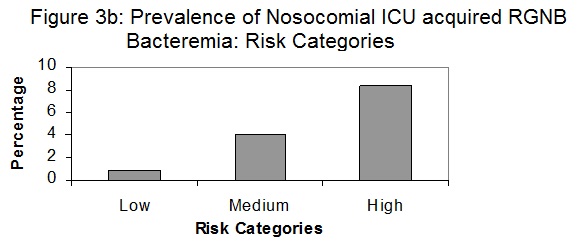

Supplement: Supplementary file 5 — Authors’ original file for figure 5 [file 12879_2014_615_MOESM5_ESM.jpeg]
